# Supplementary material for: Protein kinase A controls the hexosamine pathway by tuning the feedback inhibition of GFAT-1
Source: Nat Commun. 2021 Apr 12;12:2176. doi: 10.1038/s41467-021-22320-y (PMC8041777; doi:10.1038/s41467-021-22320-y)
Supplement: Supplementary file 1 — Supplementary Information [file 41467_2021_22320_MOESM1_ESM.pdf]

Supplementary Information for

Protein kinase A controls the hexosamine pathway by tuning the feedback inhibition of GFAT-1

Sabine Ruegenberg[1,2], Felix A.M.C. Mayr[1], Ilian Atanassov[1], Ulrich Baumann[2], Martin S. Denzel[1,3,4]*

[1]Max Planck Institute for Biology of Ageing
D-50931 Cologne, Germany

[2]Institute of Biochemistry
University of Cologne
D-50674 Cologne, Germany

[3]CECAD - Cluster of Excellence
University of Cologne
D-50931 Cologne, Germany

[4]Center for Molecular Medicine Cologne (CMMC)
University of Cologne
D-50931 Cologne, Germany

*correspondence:
martin.denzel@age.mpg.de

This file contains Supplementary Figures 1 - 5 and Supplementary Table 1

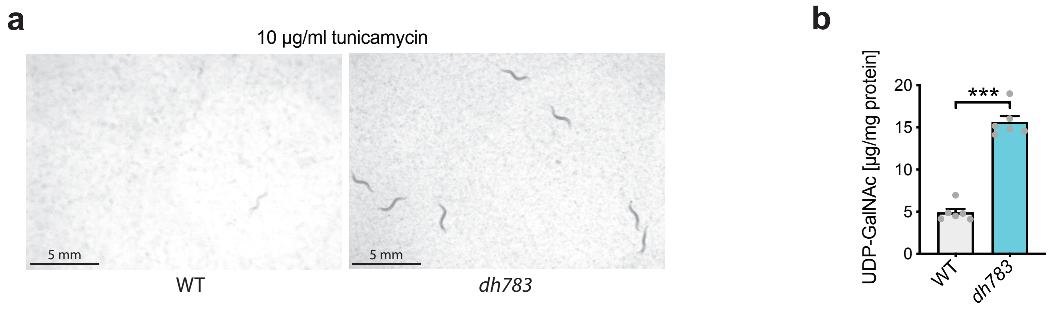

Supplementary Fig. 1 | Characterization of *gfat-1*(*dh783*) *C. elegans* mutants.

a, Representative images of N2 wild type (WT) and *gfat-1(dh783) C. elegans* 4 days post-hatch on NGM plates containing 10 µg/ml tunicamycin. **b,** UDP-GalNAc levels in wild type (WT) and *gfat-1(dh783)* animals (mean +SD, n=5, *** p<0.0001, unpaired two-sided t-test). Source data are provided as a Source Data file.

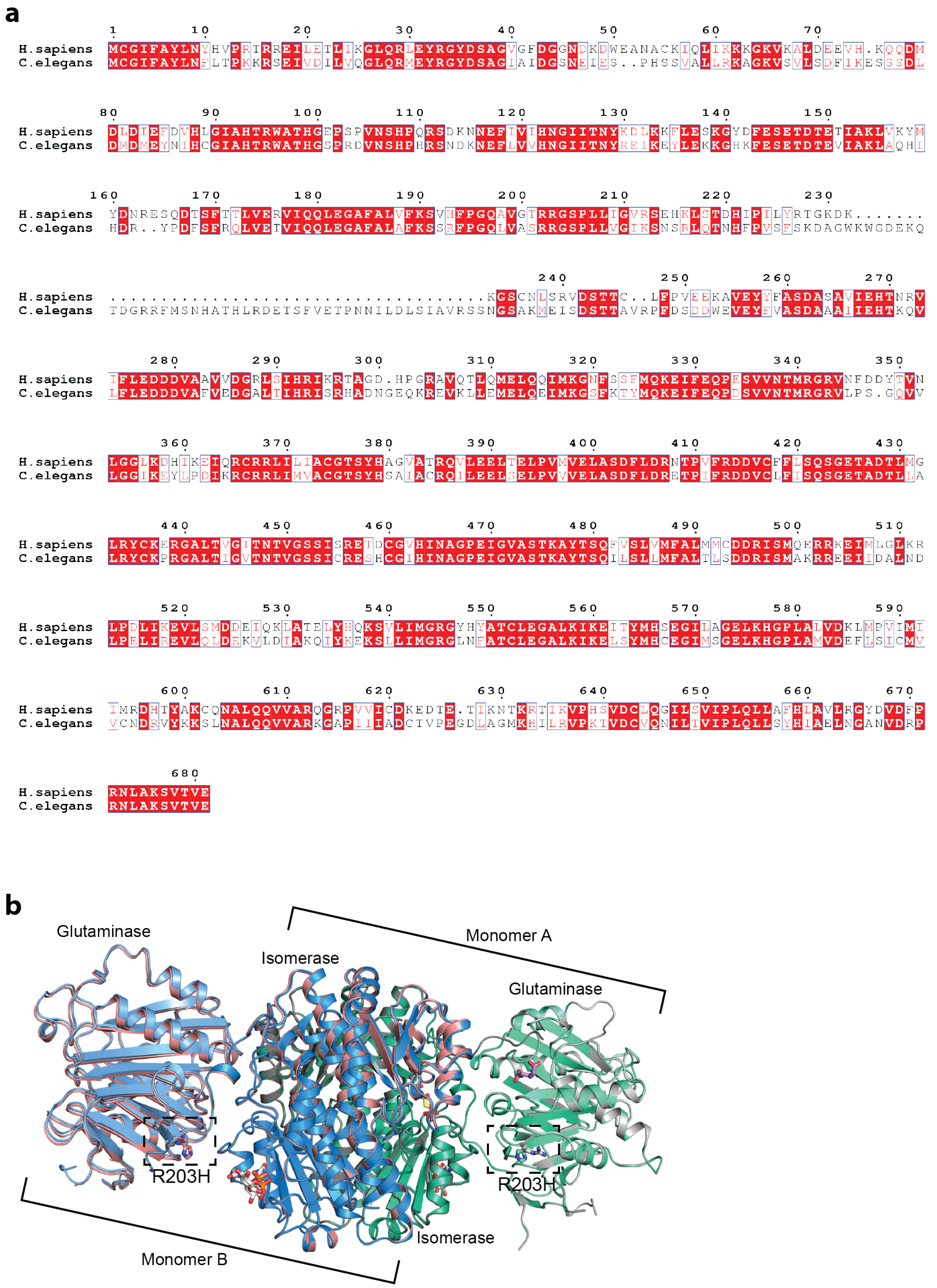

Supplementary Fig. 2 | The GFAT-1 R203H gain-of-function substitution perturbs UDP-GlcNAc feedback inhibition.

a, Protein sequence alignment of human and *C. elegans* GFAT-1. Red boxes indicate identical residues, red letters indicate similar residues. **b,** Position of the R203H mutation in the dimeric structure of GFAT-1. Proteins are presented as cartoons. Superposition of

wild type GFAT-1 (gray/blue, PDB ID 6R4E) and R203H GFAT-1 (green-cyan, salmon). Glc6P (yellow sticks), L-Glu (violet sticks), and UDP-GlcNAc (white sticks) are highlighted, as well as the position of R203H (black box). Source data are provided as a Source Data file.

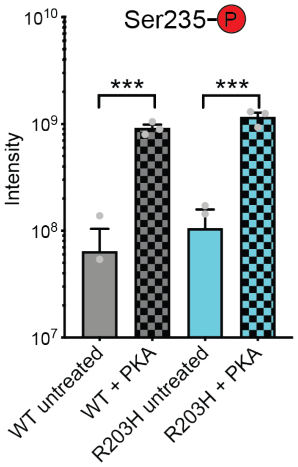

Supplementary Fig. 3 | *In vitro* PKA treatment leads to phosphorylation at Ser235 in wild type and R203H GFAT-1.

Intensity of phosphorylated peptides at Ser235 of wild type (WT, gray) and R203H (cyan) GFAT-1 before and after treatment with PKA normalized to protein abundance (mean +SEM, n=4, WT *** p=0.0002 and R203H *** p<0.0001 one-way ANOVA). Source data are provided as a Source Data file.

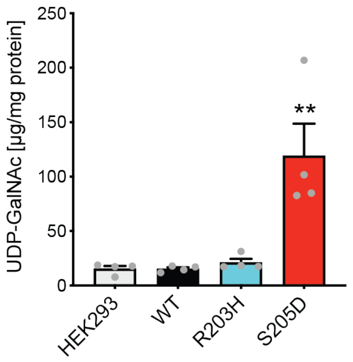

Supplementary Fig. 4 | PKA phosphorylation at Ser205 modulates UDP-GlcNAc inhibition of GFAT-1.

LC/MS measurement of UDP-GalNAc normalized to protein content presented as means +SEM with n=4, ** p=0.018 versus wild type (WT), one-way ANOVA. Source data are provided as a Source Data file.

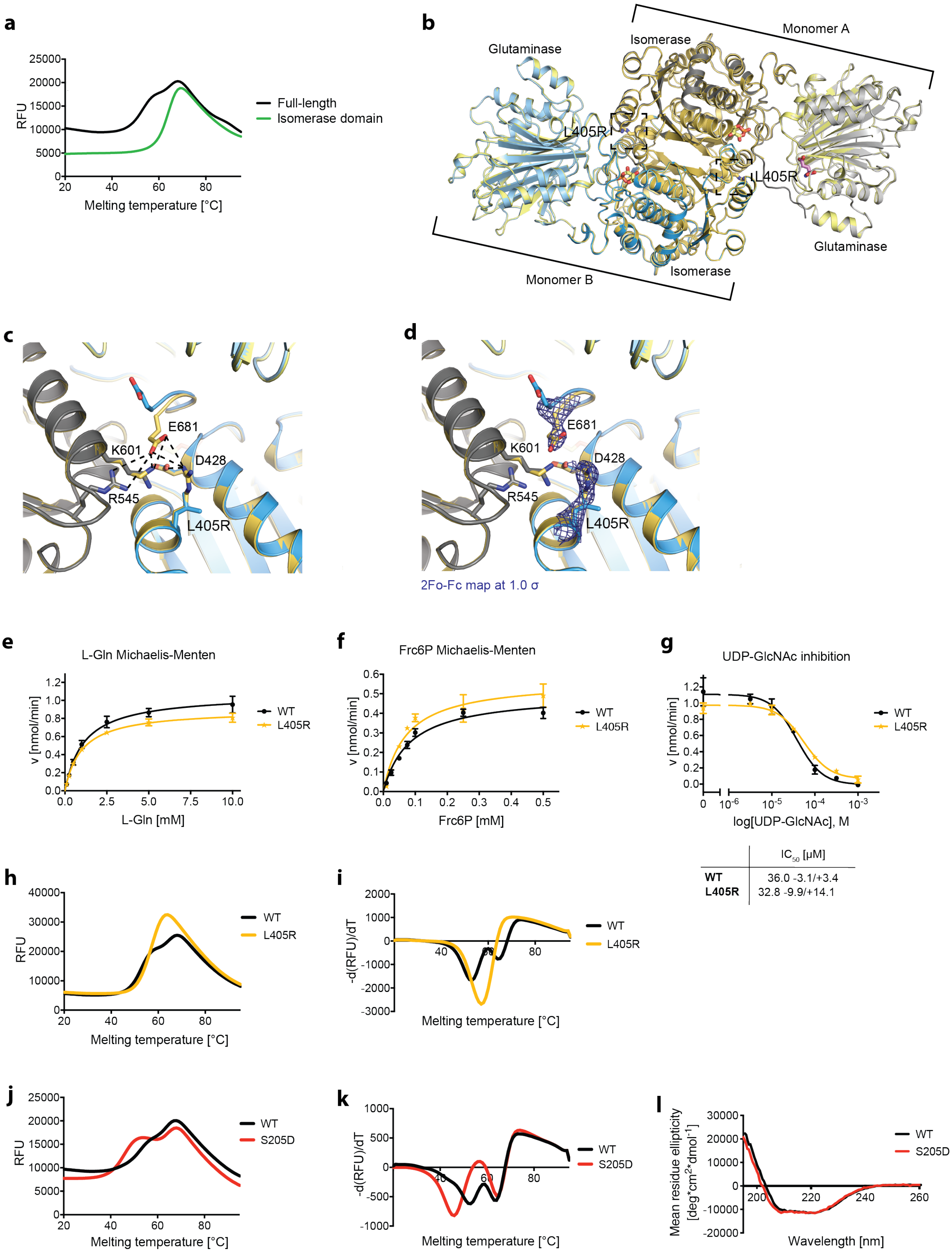

Supplementary Fig. 5 a-l | Altered domain interactions of GFAT-1 after PKA phosphorylation at Ser205. See next page for figure legend.

m

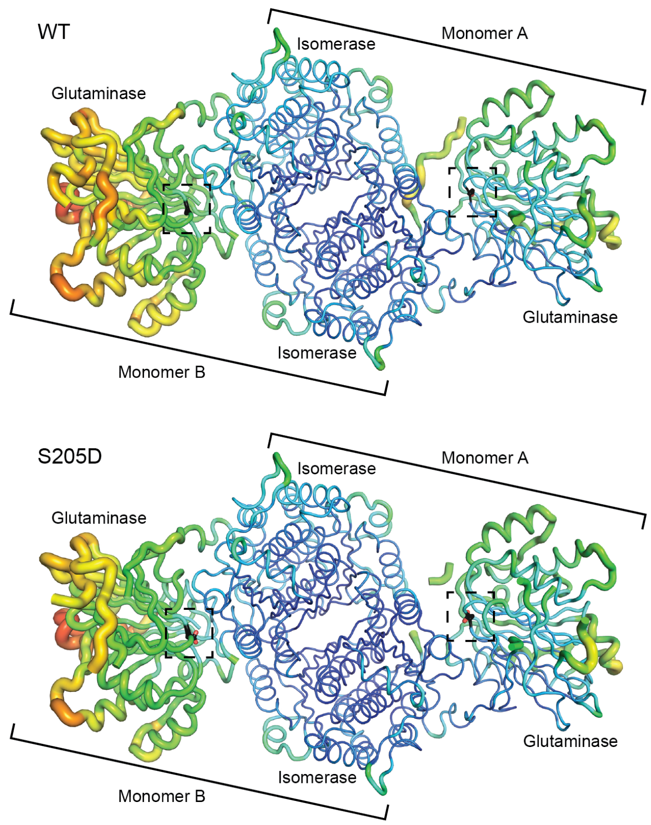

n

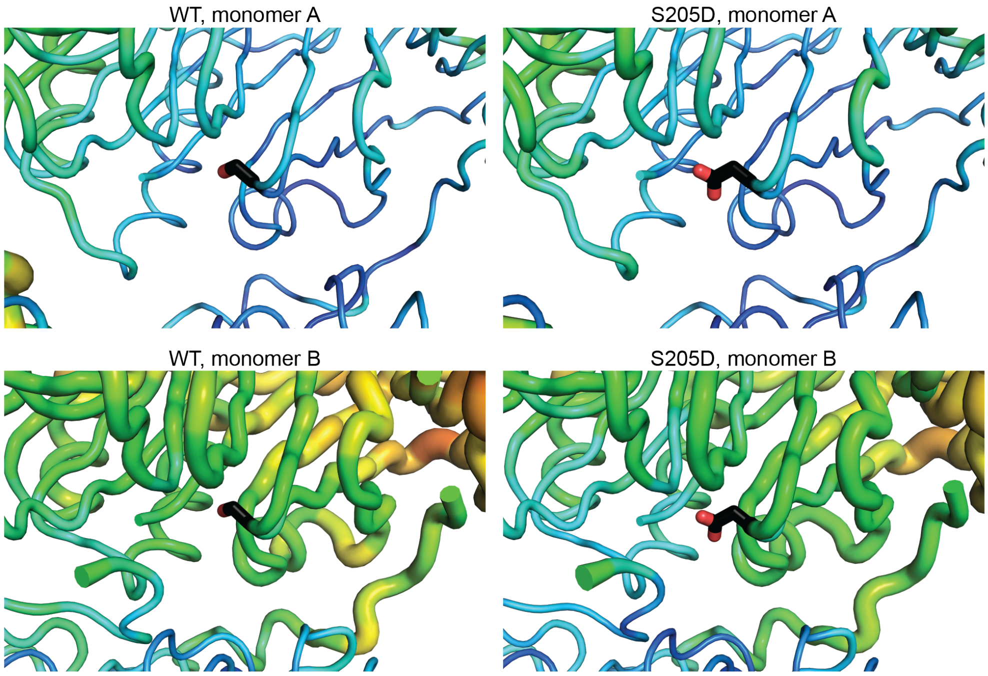

Supplementary Fig. 5 m, n | Altered domain interactions of GFAT-1 after PKA phosphorylation at Ser205.

a, Representative melting curves of full-length (black) and isolated isomerase domain (green) in standard SEC buffer without NaCl. **b-d**, Position of the L405R mutation in the

structure of GFAT-1. Superposition of the structures of wild type (monomer A gray, monomer B blue, PDB ID 6R4E) and L405R (yellow) GFAT-1 in cartoon representation. **b**, Overview of the dimers. Glc6P (yellow sticks) and L-Glu (violet sticks) are highlighted, as well as the mutation L405R (black boxes). **c**, Close-up of the interactions of L405R. **d**, Close-up of the electron density of L405R and Glu681. The 2Fo-Fc map of Arg405 and Glu681 are colored dark blue and their contour levels are at 1.5 RMSD. **e**, L-Gln kinetics of wild type (WT, black circles) and L405R (yellow stars) GFAT-1 (mean ±SEM, WT n=5, L405R n=3). **f**, Frc6P kinetics of wild type (WT, black circles) and L405R (yellow stars) GFAT-1 (mean ±SEM, WT n=5, L405R n=3). **g**, Representative UDP-GlcNAc dose response assay of wild type (WT, black circles) and L405R (yellow stars) GFAT-1 (mean ±SD, n=3). Table: IC_{50} UDP-GlcNAc values (mean ±SEM, n=3). **h**, Representative melting curves of wild type (WT, black) and L405R (yellow). **i**, Representative derivative melting curves of wild type (WT, black) and L405R (yellow). **j**, Representative melting curves of wild type (WT, black) and S205D (red). **k**, Representative derivative melting curves of wild type (WT, black) and S205D (red). **l**, CD spectra of wild type GFAT-1 (WT, black) and GFAT-1 S205D (red). **m, n,** Representation of B-factors in wild type (PDB ID 6R4E) and S205D GFAT-1 as putty cartoon. Colored from low to high values (30 to 240 $Å^2$, blue to red). **m,** Overview of the dimers. The positions of Ser205 and S205D are highlighted (sticks, dashed box). **n,** Close-up view of the area around Ser205 and S205D. Source data are provided as a Source Data file.

Supplementary Table 1 | Primers used in this study

Primer ID	Sequence (5' → 3')	Purpose
hGFAT1_W50F_for	GATAAAGATTtcGAAGCCAATGCC TGCAAAATC	Site-directed mutagenesis
hGFAT1_W50F_rev	CATTGGCTTCgaAATCTTTATCAT TGCCTCC	Site-directed mutagenesis
hGFAT1_R203H_for	CAAGGCacGGTAGCCCTCTGTTG ATTGG	Site-directed mutagenesis
hGFAT1_R203H_rev	GAGGGCTACCgtGCCTTGTGCCA ACTG	Site-directed mutagenesis
hGFAT1_S205D_for	CAAGGCGAGGTgaCCCTCTGTTG ATTGG	Site-directed mutagenesis
hGFAT1_S205D_rev	GAGGGtcACCTCGCCTTGTGCCA ACTG	Site-directed mutagenesis
hGFAT1_L405R_for	GTGACTTCCgtGACAGAAACACAC CAG	Site-directed mutagenesis
hGFAT1_L405R_rev	GTGTTTCTGTCacGGAAGTCACTT GCTAG	Site-directed mutagenesis
hGFAT1-ISO_NdeI_FOR	gagCATATGatcatgaagggcaacttcagtt catttatgc	Cloning
hGFAT1_HindIII_REV	gagAAGCTTtcactctacagtcacagatttg gcaagattc	Cloning
