# Supplementary material for: Protein kinase A controls the hexosamine pathway by tuning the feedback inhibition of GFAT-1
Source: Nat Commun. 2021 Apr 12;12:2176. doi: 10.1038/s41467-021-22320-y (PMC8041777; doi:10.1038/s41467-021-22320-y)
Supplement: Supplementary file 3 — Description of Additional Supplementary Files [file 41467_2021_22320_MOESM3_ESM.pdf]

Description of Additional Supplementary Files

File: Supplementary Data 1

Description: Phosphorylation sites in human GFAT-1 preparations
