# Supplementary material for: Protein kinase A controls the hexosamine pathway by tuning the feedback inhibition of GFAT-1
Source: Nat Commun. 2021 Apr 12;12:2176. doi: 10.1038/s41467-021-22320-y (PMC8041777; doi:10.1038/s41467-021-22320-y)
Supplement: Supplementary file 5 — Reporting Summary [file 41467_2021_22320_MOESM5_ESM.pdf]

nature research

Corresponding author(s): Martin S. Denzel

Last updated by author(s): Mar 4, 2021

Reporting Summary

Nature Research wishes to improve the reproducibility of the work that we publish. This form provides structure for consistency and transparency in reporting. For further information on Nature Research policies, see our Editorial Policies and the Editorial Policy Checklist.

Statistics

For all statistical analyses, confirm that the following items are present in the figure legend, table legend, main text, or Methods section.

n/a	Confirmed	
☐	☒	The exact sample size (*n*) for each experimental group/condition, given as a discrete number and unit of measurement
☐	☒	A statement on whether measurements were taken from distinct samples or whether the same sample was measured repeatedly
☐	☒	The statistical test(s) used AND whether they are one- or two-sided<br>*Only common tests should be described solely by name; describe more complex techniques in the Methods section.*
☒	☐	A description of all covariates tested
☒	☐	A description of any assumptions or corrections, such as tests of normality and adjustment for multiple comparisons
☐	☒	A full description of the statistical parameters including central tendency (e.g. means) or other basic estimates (e.g. regression coefficient) AND variation (e.g. standard deviation) or associated estimates of uncertainty (e.g. confidence intervals)
☐	☒	For null hypothesis testing, the test statistic (e.g. *F*, *t*, *r*) with confidence intervals, effect sizes, degrees of freedom and *P* value noted<br>*Give P values as exact values whenever suitable.*
☒	☐	For Bayesian analysis, information on the choice of priors and Markov chain Monte Carlo settings
☒	☐	For hierarchical and complex designs, identification of the appropriate level for tests and full reporting of outcomes
☒	☐	Estimates of effect sizes (e.g. Cohen's *d*, Pearson's *r*), indicating how they were calculated

Our web collection on statistics for biologists contains articles on many of the points above.

Software and code

Policy information about availability of computer code

Data collection	DA+ 2.0.0 (Swiss Light Source), Hamburg version of mxCuBE v2 (DESY), Gen5 2.01.14 (BioTek), CFX Maestro Version 3.1.1517.0823 or 4.1.2433.1219 (BioRad)
Data analysis	XDS (Mar 15, 2019 or Jan 31, 2020); phenix.phaser, phenix.refine, and phenix.elbow from the PHENIX programme package (dev_2499: ??? or 1.16-3549-000); Coot 0.8.9.1 EL; PyMOL 1.8.2.3 (Schrödinger, LLC); Prism 7 or 8 (Graphpad); MaxQuant 1.6.1.0; Skyline 19.1.0.193; CFX Maestro 3.1.1517.0823 or 4.1.2433.1219 (BioRad)

For manuscripts utilizing custom algorithms or software that are central to the research but not yet described in published literature, software must be made available to editors and reviewers. We strongly encourage code deposition in a community repository (e.g. GitHub). See the Nature Research guidelines for submitting code & software for further information.

Data

Policy information about availability of data

All manuscripts must include a data availability statement. This statement should provide the following information, where applicable:

- Accession codes, unique identifiers, or web links for publicly available datasets
- A list of figures that have associated raw data
- A description of any restrictions on data availability

Structural data reported in this study have been deposited in the Protein Data Bank with the accession codes 6ZMJ, 6ZMK and 7NDL. The mass spectrometry proteomics data have been deposited to the ProteomeXchange Consortium via the PRIDE partner repository with the dataset identifier PXD020451. The source data underlying Fig. 1c,e,f, 2a-c, 3d, 4a-f, 5b-d, 5f,g and Supplementary Fig. 1b, 2a, 3, 4, 5a, 5e-l are provided as a Source Data file. All other data supporting the presented findings are available from the corresponding authors upon request.

Field-specific reporting

Please select the one below that is the best fit for your research. If you are not sure, read the appropriate sections before making your selection.

☒ Life sciences ☐ Behavioural & social sciences ☐ Ecological, evolutionary & environmental sciences

For a reference copy of the document with all sections, see nature.com/documents/nr-reporting-summary-flat.pdf

Life sciences study design

All studies must disclose on these points even when the disclosure is negative.

Sample size	Sample sizes were chosen according to standard procedures in the field.
Data exclusions	No data were excluded from the analyses.
Replication	Activity assays and experiments with nematodes were repeated at least 3 times. All attempts at replication were successful.
Randomization	n/a as randomization is not necessary using clonal C. elegans strains.
Blinding	In C. elegans experiments, strain identity was unknown to researchers.

Reporting for specific materials, systems and methods

We require information from authors about some types of materials, experimental systems and methods used in many studies. Here, indicate whether each material, system or method listed is relevant to your study. If you are not sure if a list item applies to your research, read the appropriate section before selecting a response.

Materials & experimental systems

n/a	Involved in the study	
☐	☒	Antibodies
☐	☒	Eukaryotic cell lines
☒	☐	Palaeontology and archaeology
☐	☒	Animals and other organisms
☒	☐	Human research participants
☒	☐	Clinical data
☒	☐	Dual use research of concern

Methods

n/a	Involved in the study	
☒	☐	ChIP-seq
☒	☐	Flow cytometry
☒	☐	MRI-based neuroimaging

Antibodies

Antibodies used	GFAT1 (rb, EPR4854, Abcam ab125069), alpha-TUBULIN (ms, DM1A, Sigma T6199), rabbit IgG (gt, LifeTechnologies G21234), and mouse IgG (gt, LifeTechnologies G21040)
Validation	GFAT1: the antibody was validated with isolated human GFAT1 protein together with human and murine cell lines expressing GFAT1. According to the manufacturer, the reagent was tested with the following positive controls: IHC-P: Human testis, and Human breast carcinoma tissue; WB: Human placenta lysate, MCF7, C6, MEF, 293T, JAR and HeLa cell line lysates, Mouse heart and Mouse cerebral cortex, Rat heart tissue lysate. IP: Jurkat cells.

Eukaryotic cell lines

Policy information about cell lines

Cell line source(s)	HEK293: ATCC, Sf21: DSMZ no. ACC 119
Authentication	Cell lines were not authenticated.
Mycoplasma contamination	Cell lines were not tested for mycoplasma contamination.
Commonly misidentified lines (See ICLAC register)	No commonly misidentified cell lines were used in the study.

Animals and other organisms

Policy information about studies involving animals; ARRIVE guidelines recommended for reporting animal research

Laboratory animals	Caenorhabditis elegans strains N2 (Bristol), AA2559 [gfat-1(dh783)], MSD544 [gfat-1(syb3246)]
Wild animals	No wild animals were used in this study.
Field-collected samples	The study did not involve samples collected in the field.
Ethics oversight	No ethical approval is required for work with nematodes.

Note that full information on the approval of the study protocol must also be provided in the manuscript.
